# Rho‐associated, coiled‐coil‐containing protein kinase 1 as a new player in the regulation of hepatic lipogenesis

**DOI:** 10.1111/jdi.13051

**Published:** 2019-04-19

**Authors:** Takafumi Miyamoto, Takashi Matsuzaka, Hitoshi Shimano

**Affiliations:** ^1^ Department of Internal Medicine (Endocrinology and Metabolism) Faculty of Medicine University of Tsukuba Tsukuba‐City Ibaraki Japan

## Abstract

Rho‐associated, coiled‐coil‐containing protein kinase 1 might have applications as a potential therapeutic target for the treatment of non‐alcoholic fatty liver disease.

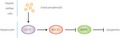

Non‐alcoholic fatty liver disease (NAFLD), an umbrella term that encompasses liver pathologies ranging from steatosis to non‐alcoholic steatohepatitis, is the most common form of chronic liver disease in the world. The prevalence of NAFLD varies from 20 to 40% in Western countries and 12–30% in Asia.

Although NAFLD represents a metabolic syndrome affecting the liver, it is also a risk factor for various diseases, including insulin resistance, hyperlipidemia, hypertension, chronic kidney disease and type 2 diabetes. Notably, NAFLD is highly prevalent among type 2 diabetes patients, and patients with NAFLD and type 2 diabetes have a higher risk of progression to cirrhosis or liver cancer compared with those who do not have type 2 diabetes.

Over the past several decades, researchers have strived to elucidate the pathogenesis of NAFLD and identify therapeutic targets, yet there are no drugs approved for treating this condition. A significant challenge to developing a NAFLD treatment is the intricacy of the disease; its progression varies among individuals with diverse genetic backgrounds, environments, microbiomes and comorbidities. Thus, further research is still required to complete the molecular puzzle that underlies the pathogenesis of NAFLD, and advance the development of effective treatments for NAFLD.

Huang *et al*.^1^ recently reported convincing evidence in the *Journal of Clinical Investigation* that hepatic rho‐associated coiled‐coil‐containing kinase 1 (ROCK1) contributes to high‐fat diet (HFD)‐induced hepatic lipogenesis through the inhibition of the energy guardian, adenosine 5´‐monophosphate‐activated protein kinase (AMPK).

ROCK1 is a serine/threonine protein kinase belonging to the protein kinase A, G and C families that are expressed in most tissues. Over the past decade, accumulating evidence has shown that ROCK1 is a multifunctional protein regulating various cellular functions, including cell motility, proliferation, cytoskeletal rearrangement and gene expression. Dysregulation of ROCK1 signaling has been found in several metabolic syndrome‐related diseases, including obesity and type 2 diabetes. However, a holistic understanding of the role of ROCK1 in the pathogenesis of these conditions remains elusive.

To investigate whether ROCK1 is associated with insulin resistance and fatty liver diseases, the authors used mice fed either a normal chow diet or HFD for several weeks. Hepatic ROCK1 levels and activity were enhanced in HFD‐fed mice compared with control mice. A similar finding was obtained in *ob*/*ob* (leptin deficient) and *db*/*db* (leptin receptor deficient) mice aged 10 weeks, as well as humans with fatty liver disease, in which the hepatic ROCK1 level was found to be increased compared with that in controls. Intriguingly, liver‐specific ROCK1‐deficient mice (*L‐ROCK1* ^*−/−*^), but not control mice, showed a decrease in bodyweight concomitant with decreasing lipid accumulation in epididymal and mesenteric fat, increased energy expenditure, and higher locomotor activity after being fed a HFD, resulting in the prevention of diet‐induced obesity. In line with these observations, *L‐ROCK1* ^*−/−*^ mice maintained on a HFD did not engender the insulin resistance often promoted by obesity.

To further support these findings, the researchers generated mice expressing a constitutively active form of ROCK1 in the liver (*L‐CA‐ROCK1*). As expected, HFD worsened obesity and insulin resistance in *L‐CA‐ROCK1* mice compared with the control mice. Although locomotor activity was not changed between control and *L‐CA‐ROCK1* mice, energy expenditure was demonstrably decreased in *L‐CA‐ROCK1* mice.

Obesity is closely associated with the development of NAFLD. Indeed, 70% of overweight individuals, 70% of diabetes patients and up to 90% of morbidly obese individuals have been found to have NAFLD^2^. In principle, hepatic lipid accumulation results from disruption of the orchestrated balance of lipid metabolism, which is governed by a physiological quartet: uptake of circulating fatty acids, fatty acid oxidation, triglyceride export as very low‐density lipoprotein and hepatic de novo lipogenesis (DNL). Looking at lipid metabolism in the liver of bodyweight‐matched *L‐ROCK1* ^*−/−*^ mice and control mice fed with a HFD, the authors concluded that hepatic ROCK1 plays a pivotal role in obesity‐induced hepatic steatosis through upregulation of lipogenesis‐related genes that promote hepatic DNL. Consistently, the expression levels of genes encoding lipogenic enzymes were increased in *L‐CA‐ROCK1* mice, but not control mice, after being fed a HFD. Of note, unlike DNL, the regulation of fatty acid oxidation, fatty acid uptake and triglyceride secretion was not altered by hepatic ROCK1 activity, as judged by the expression level of related genes and the serum lipid profile in *L‐ROCK1* ^*−/−*^ and *L‐CA‐ROCK1* mice.

De novo lipogenesis, a fundamental function of the liver, is the biochemical process of fatty acid synthesis from acetyl‐coenzyme A obtained from carbohydrate metabolism (e.g., glycolysis). In general, dietary carbohydrates not stored as glycogen or oxidized in response to the immediate energy demand are to be subjected to DNL. Therefore, excess carbohydrate intake could prime DNL. Although DNL is closely intertwined with the pathogenesis of NAFLD, this relationship varies widely depending on the experimental conditions (e.g., population, method of hepatic steatosis assessment etc.). In addition, the issue of whether DNL is attributable to HFD remains controversial^3^. Furthermore, although it is known that obesity is the strongest risk factor for NAFLD, DNL is not an integral contributor to obesity. Therefore, it is likely that obesity, NAFLD and DNL represent vertexes of a triangle, but the connections among the vertexes are not well understood. However, as shown by Huang *et al*.^1^, these connections could potentially be clarified by understanding the role of ROCK1.

How hepatic ROCK1 potentiates DNL in HFD‐fed mice remains a fascinating biological question. The researchers focused on the endocannabinoid system to attempt to address this question. Endocannabinoids are endogenous agonists of cannabinoid receptors (CB1R and CB2R) that elicit a broad spectrum of physiological responses. So far, two arachidonic acid derivatives, anandamide and 2‐arachidonoylglycerol, have been identified as endocannabinoids. Osei‐Hyiaman *et al*.^4^ previously reported that hepatic CB1R is involved in hepatic DNL, leading to the hypothesis that the lipogenic activity of hepatic ROCK1 is regulated by the endocannabinoid system. Examining ROCK1 activity in response to the endocannabinoid system *in vitro* and *in vivo* showed that this system contributes upstream signaling components that lead to the activation of ROCK1 followed by hepatic DNL.

The apparent role of the endocannabinoid system in ROCK1‐mediated hepatic DNL ties into the involvement of AMPK in this process, as CB1R and AMPK are part of a signaling network in the regulation of hepatic DNL. However, the molecular mechanisms underlying the CB1R–AMPK axis remain unclear. Huang *et al*.^1^ successfully showed that ROCK1 fills a gap between CB1R and AMPK in the signaling pathway. More specifically, endocannabinoids known to be secreted from hepatic stellate cells upon HFD activate ROCK1 in hepatocytes, resulting in the inhibition of AMPK, followed by the upregulation of lipogenesis.

The research summarized here undoubtedly extends our understanding of NAFLD pathogenesis (Figure 1). However, further research is still required to fully clarify the underpinnings of this process.

**Figure 1:**
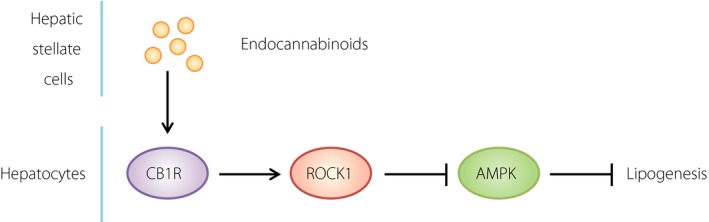
Proposed model of rho‐associated coiled‐coil‐containing kinase 1 (ROCK1)‐mediated lipogenesis in the liver. Upon high‐fat feeding, the endocannabinoids secreted from hepatic stellate cells provoke ROCK1 activation through a cannabinoid receptor on the surface of hepatocytes. Once ROCK1 is activated, adenosine 5´‐monophosphate‐activated protein kinase (AMPK) was shut down through an unknown mechanism, leading to potentiation of lipogenesis in the hepatocytes. CB1R, cannabinoid receptor 1..

First, what lipid species were predominantly accumulated through ROCK1‐mediated lipogenesis in the liver? In the current conceptual framework, a causative pathogenic driver of NAFLD involved an accumulation of toxic lipid species, which provoke endoplasmic reticulum stress and hepatocellular injury, which then predispose the liver to cirrhosis and hepatocellular carcinoma after fibrogenesis and genomic instability. Elucidating the specific toxic lipid species involved will likely reveal new therapeutic opportunities, where changing the lipid quality as opposed to the quantity can help prevent NAFLD.

Second, it is as yet unknown how the endocannabinoids–ROCK1 axis inhibits AMPK. Huang *et al*.^1^ did not feel that ROCK1 per se is a novel direct AMPK inhibitor, as they could not identify an interaction between ROCK1 and AMPK. Although this suggests indirect suppression of AMPK by ROCK1, the underlying molecular mechanisms require further investigation. One possibility is a rearrangement of AMPK dynamics. To date, a growing body of evidence suggests that, under certain conditions, physiological input encoded in the pattern of spatiotemporal dynamics of AMPK seems to be crucial in determining the specific downstream AMPK function^5^. AMPK dynamics can be altered by various factors, including the organelle morphology, number and size of signaling platforms on membrane, and molecular congestion in the cytosol, all of which are attributed, at least partially, to the lipid profile. Therefore, the molecular environment created by ROCK1 might negatively regulate AMPK, leading to lipogenesis. Further research is required to clarify this process.

Finally, it is unclear why hepatic ROCK1 deficiency prevents HFD‐induced obesity in mice. The study authors hypothesize that hepatokines might be involved. Another possibility is a reorganization of the blood metabolome, whose functions include not only contributing to the building blocks of a cell, but also producing signaling cues to regulate various signaling pathways. Disentangling the mechanisms underlying the prevention of obesity through ROCK1 deficiency will provide further insight into critical aspects of cell biology, and might also lead to personalized medicine options for the treatment or prevention of NAFLD.

## Disclosure

The authors declare no conflict of interest.
